# Toward a Better Regeneration through Implant‐Mediated Immunomodulation: Harnessing the Immune Responses

**DOI:** 10.1002/advs.202100446

**Published:** 2021-06-12

**Authors:** Ben Zhang, Yingchao Su, Juncen Zhou, Yufeng Zheng, Donghui Zhu

**Affiliations:** ^1^ Department of Biomedical Engineering Stony Brook University Stony Brook New York 11794 USA; ^2^ Department of Materials Science and Engineering College of Engineering Peking University Beijing 100871 China

**Keywords:** foreign body response, immunomodulation, macrophage, neutrophil, tissue engineering

## Abstract

Tissue repair/regeneration, after implantation or injury, involves comprehensive physiological processes wherein immune responses play a crucial role to enable tissue restoration, amidst the immune cells early‐stage response to tissue damages. These cells break down extracellular matrix, clear debris, and secret cytokines to orchestrate regeneration. However, the immune response can also lead to abnormal tissue healing or scar formation if not well directed. This review first introduces the general immune response post injury, with focus on the major immune cells including neutrophils, macrophages, and T cells. Next, a variety of implant‐mediated immunomodulation strategies to regulate immune response through physical, chemical, and biological cues are discussed. At last, various scaffold‐facilitated regenerations of different tissue types, such as, bone, cartilage, blood vessel, and nerve system, by harnessing the immunomodulation are presented. Therefore, the most recent data in biomaterials and immunomodulation is presented here in a bid to shape expert perspectives, inspire researchers to go in new directions, and drive development of future strategies focusing on targeted, sequential, and dynamic immunomodulation elicited by implants.

## Graphical abstract

Implant‐mediated immunomodulation represents an emerging research topic in the field of tissue engineering, and is a new promising direction to achieve better tissue regeneration. The underlying mechanism of immune response and its influence on tissue repair, immunomodulatory strategies, recent in vivo applications of implant‐based immunomodulation to improve tissue regeneration are summarized, and current challenges and future prospect are also discussed.

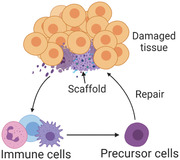

## 1 Introduction

Tissue/organ dysfunction suffering from aging,^[^ ^1^, ^2^ ^]^ injuries,^[^ ^3^, ^4^, ^5^, ^6^, ^7^ ^]^ and diseases^[^ ^8^, ^9^ ^]^ presents a serious threat to human health, and leads to a high demand for organ transplantation.^[^ ^10^, ^11^, ^12^ ^]^ Organ transplantation is one of the strongest medical field practices to combat this problem, but is drastically limited by supply, despite a steadily increasing number of donor population. While promising, unfortunately we are still far away from meeting the demand.^[^ ^13^, ^14^ ^]^ As a result, tissue repair/regeneration medicine emerges as a promising alternative for clinicians to turn to address this problem.^[^ ^15^, ^16^ ^]^ This strategy relies on renewal and growth of patients’ own organs, which can avoid the potential disease transmission and long‐term immune rejection from transplanted ones.^[^ ^17^ ^]^ Recruiting, proliferation and differentiation of stem and progenitor cells, with the help of resorbable scaffolds that serve as extracellular matrix (ECM) to support cell activities, following appropriate physiological processes have naturally been considered the key points for tissue regeneration.^[^ ^18^, ^19^ ^]^ On the other hand, increasing evidence demonstrated that immune system also plays an important role to achieve successes for tissue regeneration, directly or indirectly.^[^ ^20^, ^21^ ^]^ Immune response to foreign body initiates complex host defense cascade and often drives to scarring and fibrosis, and results in impaired tissue repair and failure of organ function.^[^ ^22^, ^23^ ^]^ Therefore, a multitude of efforts (e.g., delivery of immunosuppressants) have been dedicated to attempting to avoid overreacting immune response in order to achieve desired tissue regeneration.^[^ ^24^, ^25^ ^]^ However, recent studies have demonstrated that some type of immune cells can resolve immune reaction and contribute to a healthy tissue repair.^[^ ^23^, ^26^ ^]^ To this end, manipulation of leukocytes via appropriate physical, chemical, and biological cues to reduce harmful immune reaction while enhancing beneficial cell activities could promote a better healing. In addition to these immunomodulatory factors, biomaterial implants that serve as the delivery vehicle are also crucial to achieve successful tissue regeneration. They can preserve tissue architecture, offer biocompatible supports for cell attachment, proliferation and differentiation, protect cargos from damaging, and locally release drugs in a controlled manner to guide tissue regeneration. Moreover, biomaterial implants are demonstrated to have influence on immune response even in the absence of immune‐stimulating signals. Considering the feasible variety of chemical and physical modification of biomaterials, biomaterial implants provide promising potentials for tissue regeneration. Based on these considerations, this review summarizes the latest data and discusses how to improve tissue regeneration through immunomodulation. First, we will establish foundation in covering basics on immune reactions to tissue damages, followed by immunomodulatory approaches used to regulate immune responses, and ultimately, the applications of biomaterial implants on regenerations of different tissues harnessing immunomodulatory strategy.

## 2 Innate and Adaptive Immune System

**Figure 1:**
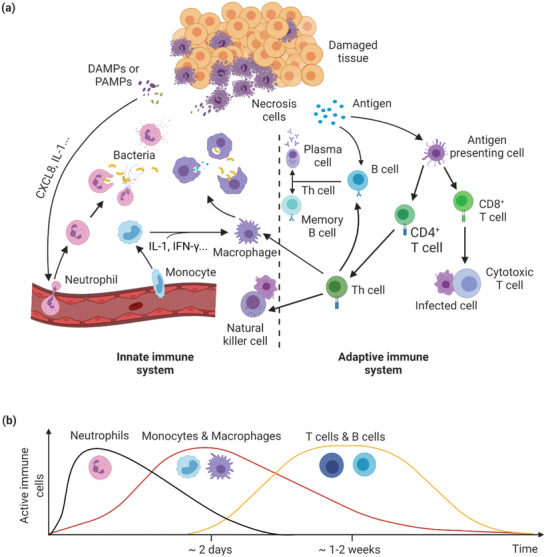
Immune response after injury. a) Overview of innate and adaptive immune system participates in immune response. b) Kinetics of activated immune cells involved in immune response. DAMPs: Damage‐associated molecular patterns; PAMPs: Pathogen‐associated molecular patterns; CXCL8: C–X–C motif ligand 8; IL‐1: Interleukin‐1; IFN‐*γ*: Interferon‐*γ*; Th cell: T helper cell.

**Figure 2:**
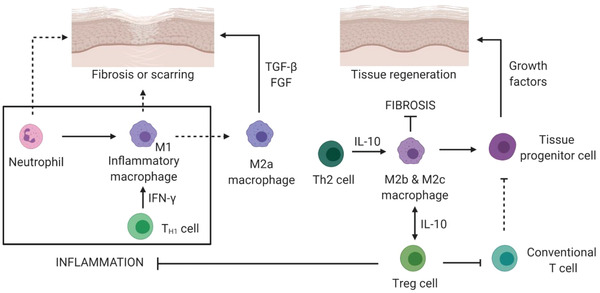
Immune response during tissue regeneration. Over inflammatory reaction and sustained inflammation lead to impaired/fibrotic tissue formation. Anti‐inflammatory modulation helps to tissue regeneration. T_H1_ cell: T helper type 1 cell; Treg cell: Regulatory T cell.

The immune system provides defense against risk/invasion through a well‐orchestrated inflammatory cascade, which involves a series of sequential processes including threat detection, danger clearance and homeostasis restoration.^[^ ^27^, ^28^ ^]^ After injury, necrotic cells or fragments from damaged ECM can release some pro‐inflammatory molecules, which are referred to as damage‐associated molecular patterns (DAMPs).^[^ ^29^ ^]^ Similarly, molecular signals released from invaded bacteria, funguses and viruses are called pathogen‐associated molecular patterns (PAMPs).^[^ ^30^ ^]^ Both DAMPs and PAMPs can induce a local inflammation and promote tissue‐resident immune cells to secret inflammatory factors that would recruit more immune cells from the circulating system to the damage site. On the basis of response kinetics and function, immune system is classified into two different types, innate and adaptive immunity (**Figure** **1**). Innate immune system is considered as the first responder to risk, which includes neutrophils, monocytes, and macrophages. When these innate immune cells fail to defeat risk, they can mobilize adaptive immunity of B cells and T cells, which can specifically eliminate the encountered threat.^[^ ^31^ ^]^ In addition to removing debris of apoptotic cells, ECM fragments and pathogens during inflammation, immune cells are also involved in the process of tissue repair/regeneration (**Figure** **2**). They can promote/resolve inflammation to impair/help stem/tissue cell proliferation and differentiation, leading to scarring or tissue restoration. Therefore, in order to achieve the desired tissue regeneration, it is very important to understand the underlying mechanism of immune response of different immune cells.

### 2.1 Immune Cells and Immune Responses

#### 2.1.1 Neutrophils

Neutrophils belong to polymorphonuclear family and originate from bone marrow stem cells. They are innate immune cells and usually considered as the first ones migrating toward the damage site in response to injury or external invasion.^[^ ^32^ ^]^ Histamine and cytokines released from damaged tissue resident cells, as well as pathogen‐related bio‐signals can result in vasodilation, increase permeability of blood vessels, and stimulate neutrophils, driving them transmigration to the injury region from circulating system.^[^ ^33^, ^34^ ^]^ Comparing to other cells with a large spherical nucleus, the multi‐lobe nuclear morphology afford neutrophils high flexibility and enable them the better ability to migrate through blood vessels and narrow gaps between tissue cells and ECM.^[^ ^35^ ^]^ Once at the injury site, neutrophils are immediately engaged in eliminating the perceived threat. They can either secrete bactericidal contents and proteases to destroy pathogens, or produce neutrophil extracellular traps (NETs) to engulf bacterial.^[^ ^36^, ^37^ ^]^ NETs, made of extracellular proteins and chromatin, are specific networks extruded from neutrophils. The release of NETs usually comes with a cell death process called NETosis and can be triggered by reactive oxygen species (ROS) and microbial cues.^[^ ^38^ ^]^ Formation of NETs begins with loss of nuclear lobules and cellular polarization, followed by chromatin decondensation and cell membrane rupture, and then NETs release. Due to the high affinity and local high concentration of antibacterial components, NETs can efficiently trap and kill pathogens.^[^ ^39^ ^]^ However, because of dangerous cytokines secreted and its nonspecifically attack, neutrophils have been considered to impair tissue healing process^[^ ^40^ ^]^ and accelerated wound closure was observed with neutrophil depletion.^[^ ^41^ ^]^ On the other hand, neutrophils can also secret vascular endothelial growth factor (VEGF),^[^ ^42^ ^]^ growth factors,^[^ ^43^, ^44^ ^]^ and matrix metalloproteinases (MMPs)^[^ ^45^ ^]^ to promote angiogenesis, stimulate cell proliferation and ECM remodeling. In addition, apoptosis of neutrophils and subsequent phagocytosis by macrophages can trigger anti‐inflammatory activities and help to resolve inflammation.^[^ ^46^, ^47^ ^]^ Therefore, neutrophils are more than the conventionally defined suicidal killers and they play an important role in immune regulation as well. Neutrophils can produce interferons (IFN‐*γ*) to recruit macrophages, activate dendritic and natural killer (NK) cells through toll‐like receptor 9 (TLR9) pathway.^[^ ^32^, ^48^, ^49^ ^]^ Reciprocally, other immune cells such as macrophages and T cells can also regulate neutrophil production and migration.^[^ ^32^, ^50^ ^]^ Through TLR9/myeloid differentiation factor 88 (MyD88) pathway, tissue resident macrophages can express neutrophil chemoattractants such as (C–X–C motif) ligand (CXCL)2 and CXCL5 that promote neutrophil recruitment,^[^ ^51^ ^]^ and the accumulation of neutrophils is usually considered detrimental for tissue regeneration.^[^ ^52^ ^]^ In addition to inducing neutrophil apoptosis, regulatory T cells (Treg) can promote interleukin (IL)‐10 (IL‐10) and transforming growth factor (TGF‐*β*1) expression but inhibit IL‐6 production from neutrophils,^[^ ^53^ ^]^ generating an anti‐inflammatory condition in favor of tissue repair.

#### 2.1.2 Monocytes and Macrophages

Tissue resident macrophages mostly come from yolk sac during embryogenesis. They play critical role during tissue development and help tissue homeostasis.^[^ ^54^ ^]^ After injury, a large number of circulating monocytes are recruited to the damage site via sensing chemokine and cytokine signals. Along with resident macrophages, these immune cells undergo remarkable phenotypic and functional changes as they participate in inflammation and subsequent tissue healing process.^[^ ^55^, ^56^ ^]^ As progenitors of macrophages and dendritic cells (DCs), monocytes are a population of heterogeneous cells fund in the bone marrow, blood and spleen. They exhibit distinct surface makers, cell activities, and functions depending on different animals and subsets.^[^ ^57^, ^58^ ^]^ In mice, inflammatory monocytes (IMs) highly express lymphocyte antigen‐6 complex (Ly6C)^high^ and C—C chemokine receptor 2 (CCR2),^[^ ^59^ ^]^ while cluster of differentiation (CD)14^+^CD16^−^ marks a typical human subset.^[^ ^60^ ^]^ IMs are recruited to inflammation sites with the help of integrin and chemokine receptors such as CCR2 and CCR5 that can be attracted by an inflammatory cytokine monocyte chemoattractant protein (MCP).^[^ ^61^ ^]^ After injury, IMs promote inflammation and peak their concentration at ≈48 h.^[^ ^62^ ^]^ On the contrary, anti‐inflammatory monocytes (AMs) contribute to resolve inflammation. They promote matrix modeling, angiogenesis and prevention of fibrosis, through secretion of cytokines such as TGF‐*β* and IL‐10.^[^ ^63^, ^64^ ^]^ AMs are characterized with cell surface makers of Ly6C^low^, C‐X3‐C motif chemokine receptor 1 (CX3CR1)^high^, and CCR2^−^ in mice, and CD14^low/−^CD16^+^ in human.^[^ ^65^ ^]^ Usually, AMs have a longer half‐life time than that of IMs and can be derived from IMs under certain stimulus.

Similar to monocytes, macrophages also have distinct subsets to promote and resolve inflammation. The pro‐inflammatory phenotype, which is called M1 macrophages, can be triggered under an inflammatory environment through cytokines such as IFN‐*γ* and tumor necrosis factor (TNF‐*α*), and chemokine lipopolysaccharide.^[^ ^66^ ^]^ M1 macrophages are well‐known as scavengers which secret reactive chemicals, phagocytize apoptotic neutrophils, necrotic tissue fragments, and clear pathogens.^[^ ^67^ ^]^ On the other hand, they also produce growth factors such as VEGF and fibroblastic growth factor (FGF).^[^ ^68^ ^]^ Over‐activation or sustained mobilization of M1 could impair tissue healing and lead to tissue damage.^[^ ^69^ ^]^ Different from M1 macrophages, anti‐inflammatory M2 macrophages are considered to help tissue homeostasis.^[^ ^70^, ^71^ ^]^ This phenotype of macrophages includes M2a, M2b, and M2c, distinguished by their cell surface markers, activators, and cytokine expression.^[^ ^72^ ^]^ M2a macrophages can be activated with IL‐4 and/or IL‐13. They are involved in matrix remolding by expressing relevant cytokines to promote formation of collagen and fibrous tissue, leading to wound contraction and closure.^[^ ^73^, ^74^ ^]^ M2b and M2c can secret IL‐10, which is an immunoregulatory cytokine, helping to suppress scarring formation.^[^ ^75^, ^76^ ^]^ This cytokine (IL‐10) can also regulate development of Treg that resolve inflammation.^[^ ^77^ ^]^ Although M2 macrophages are thought to help tissue healing, it should be noted that persistent activation of this phenotype could lead to detrimental tissue repair and pathological fibrosis.^[^ ^78^ ^]^

#### 2.1.3 T cells and Other Immune Cells

T cells belong to adaptive immune system and plays critical role in specific immune response. Like macrophages, there are several T cell subsets with distinct functionalities triggered by different chemokines and cytokines.^[^ ^79^, ^80^ ^]^ Cytotoxic CD8^+^ T cells are found to inhibit osteogenic process and bone healing was accelerated after depletion of CD8^+^ T cells.^[^ ^81^ ^]^ For CD4^+^ T cells, the impact on tissue healing differs based on their phenotypes. IFN‐*γ*, which can be secreted by T helper type 1 (Th1) cells, is found to inhibit bone formation in a mouse model.^[^ ^82^ ^]^ Contrarily, Th2 cells helps to resolve inflammation through regulation on anti‐inflammatory M2 macrophages, which are triggered by cytokines such as IL‐4 and IL‐10.^[^ ^83^, ^84^ ^]^ Treg cells, which is characterized by the cell marker Forkhead box P3, can suppress over‐active and regulate uncontrolled inflammation to facilitate immune system homeostasis.^[^ ^85^, ^86^ ^]^ By secreting anti‐inflammatory cytokines IL‐10 and TGF‐*β*, Treg cells can prevent neutrophils to produce IL‐6, thus reduce inflammation and induce neutrophil apoptosis.^[^ ^87^, ^88^ ^]^ In addition, Treg cells can regulate transmigration of neutrophils to injury site.^[^ ^89^ ^]^ Treg cells also help to suppress monocyte to secret inflammatory cytokines and promote macrophages to polarize toward M2 phenotype.^[^ ^90^, ^91^, ^92^ ^]^ As aforementioned, conventional T cells secrete cytokines such as INF*γ* and TNF‐*α* to sustain inflammation. On the contrary, Treg cells produce anti‐inflammatory cytokines to control activity of these conventional T cells.^[^ ^93^, ^94^ ^]^ As a result of suppression on conventional CD4^+^ T cells, improved osteoblast differentiation was observed with the help of Treg cells.^[^ ^95^ ^]^ In addition to regulation on other immune cells, Treg cells are also found to have a directly impact on tissue cells. Active Treg cells can secret growth factor Amphiregulin to promote muscle healing.^[^ ^96^ ^]^ *γδ*T cells are another T cell subtype. These types of T cell also serve an important role in tissue repair. They can secret growth factors and cytokines such as insulin‐like growth factor (IGF‐1), FGF‐1a, and IL‐17A to help tissue cells homeostasis and immune cell recruitment.^[^ ^97^, ^98^, ^99^ ^]^ Other studies also provide data which supports inefficient wound healing was observed in the absence of *γδ*T cells.^[^ ^100^, ^101^ ^]^

Other immune cells such mast cells^[^ ^102^, ^103^ ^]^ and dendritic cells^[^ ^104^ ^]^ are also involved in tissue healing process. Mast cells can secret a variety of effectors to recruit eosinophils and monocytes and promote inflammation.^[^ ^105^, ^106^ ^]^ On the other hand, they were found to produce anti‐inflammatory cytokines to resolve inflammation.^[^ ^107^ ^]^ Dendritic cells are best‐known for their antigen‐presenting role to contribute to adaptive immune response.^[^ ^108^ ^]^ However, they can secrete IFN‐*γ* and delayed wound closure was observed in mice depleted with dendritic cells.^[^ ^109^ ^]^ B cells belong to family of adaptive immune system and their main role during immune response is to present antigens and secret antibodies. Some studies found B cells can regulate neutrophil infiltration, increase production of growth factors, and suppress MMP2 expression to promote wound healing.^[^ ^110^ ^]^

### 2.2 Interactions between Immune Cells and Implants

Immediately after implantation, a variety of plasma proteins such as albumin, fibronectin, vitronectin, and others will deposit on implant surface. These absorbed proteins have important impacts on the recruitment, adhesion, and activity of immune cells to implant site.^[^ ^28^ ^]^ Released from blood coagulation that is deposited on implants, platelet‐derived TGF‐*β*, CXCL2, and CXCL8 can attract neutrophils from circulating system,^[^ ^111^ ^]^ and integrins expressed by neutrophils help to bind onto implant surface coated with protein layer. This kind of binding can promote neutrophil activation and initiate inflammatory response which further induce the recruitment of immune cells.^[^ ^112^ ^]^ Prolonged activation of neutrophils results in chronic inflammation and delayed tissue healing. On the other hand, neutrophils can also contribute to tissue regeneration through growth factor secretion, helping mesenchymal stem cells (MSCs) remodeling and resolving inflammation via apoptosis as discussed before. Analogous to neutrophils, release of chemoattractants such as TGF‐*β*, CXCL4, and leukotriene from platelets and clot can guide monocytes/macrophages to implant site.^[^ ^113^, ^114^ ^]^ The absorbed proteins on implant promote macrophage adhesion with the help of integrin. Accumulation of macrophages at implant site leads to further chemoattractant secretion that recruits additional macrophages and activated macrophages tend to phagocytose biomaterials.^[^ ^115^, ^116^ ^]^ These inflammatory macrophages at early stage produce a series of toxic materials such as ROS, degradative enzymes, and acid that are considered detrimental for tissue healing.^[^ ^117^ ^]^ In a later inflammation stage, macrophages polarize to M2‐type phenotype that helps to tissue healing through expression of anti‐inflammatory cytokines. Dendritic cells can sense biomaterials through toll‐like receptors upon activation by ligand moieties on adsorbed protein layer deposited on implants. With the help of integrin, dendritic cells are capable to adhere to fibronectin.^[^ ^118^ ^]^ Albumin can stimulate dendritic cells to produce anti‐inflammatory cytokine IL‐10, helping inflammation resolution and tissue healing. On the contrary, vitronectin resulted in IL‐12p40 expression from dendritic cells that correlated with CD4+ T cell proliferation,^[^ ^119^ ^]^ which can impair tissue restoration. Overall, adsorbed proteins on implants play a great role to mediate the interactions between implants and immune cells, thus modification of surface properties can result in changes of protein deposition on biomaterials and consequently modulate responses from immune cells.

## 3 Strategies to Regulate Immune Response

As articulated in the above sections, the immune system plays critical role in tissue repair. Therefore, it is imperative and seemingly necessary to manipulate and govern the immune response within tissue damage as a new focus to promote efficient tissue healing. In recent decades, many efforts have been tried to explore strategies on how to control immune cell polarization and cell behavior toward an anti‐inflammatory state, which promote tissue regeneration and function restoration. In the following context, several commonly used methods for immunomodulation, which are based on different type of stimuli and the corresponding mechanism will be discussed.

### 3.1 Physical Signals

**Figure 3:**
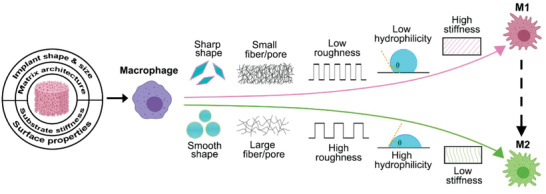
Physical signals, including the implant shape, substrate stiffness, and different surface properties, can cause the various foreign body response. While conditions like sharp implant shape, high stiffness of the substrate, low roughness and hydrophilicity, and small fiber/pore can trigger the strong inflammatory response (M1 subtype of macrophages), other conditions like smooth implant shape, low stiffness of the substrate, high roughness and hydrophilicity, and large fiber/pore are associated with the anti‐inflammatory response (M2 subtype of macrophages).

Once an implant is placed into the human body, the immune system will inherently seek to interfere with it. Physical features of the implant can prompt signals and indicators leading to the primary cause for the following immune response. Most prior studies concerning the physical signals were focused on macrophages (**Figure** **3**).

The shape and size of implants have been reported to influence foreign body response. As a consequence of implant movement, a sharp shape could lead to great tissue response.^[^ ^120^ ^]^ Hydroxyapatite (HAp), a commonly utilized biomaterial for bone regeneration, has similar chemical components as bone mineral and can be formed into different shapes and sizes. HAp in the form of rounded particles was reported to resolve inflammation at a faster rate than that of sharp‐edged particles when implanted in buccal soft tissue pouches of beagle dogs.^[^ ^121^ ^]^ Meanwhile, it was found that the innate immune response of mice is dependent on both morphology and size of HAp particle, which is through activation of the nod‐like receptor family pyrin domain containing 3 (NLRP3) inflammasome and IL‐1*β* secretion. Enhanced cytokine secretion was observed with needle‐shaped and small HAp particles, leading to a robust inflammatory response, while larger spherical particles with smooth surface did not. In addition, the shape of HAp particles causes influence on patterns of innate immune cell recruitment, for example, needle‐shaped particles trigger the increasing recruitment of neutrophils and eosinophils compared to spherical particles,^[^ ^122^ ^]^ which can be attributed to more activating proteins absorbed on biomaterials with irregular shape that have higher surface area.^[^ ^111^ ^]^

The influence of the geometry of implants on the foreign body responses was also studied in vivo. Spheres with 1.5 mm and above in diameter across a broad spectrum of materials significantly abrogated foreign body reactions and fibrosis, when compared with smaller ones after implantation into rodent and non‐human primate animals. Fibrotic tissue collected from mice implanted of the 0.5 mm spheres presented increasing expression of markers associated with all three macrophage phenotypes. On the other hand, in the case of 1.5 mm spheres, the expression of macrophage markers associated only with classical and wound‐healing phenotypes.^[^ ^123^ ^]^ However, the influence of implant size on foreign body response is still elusive and sometimes controversial. A study found that the larger size of materials composed of polyurethane, silicone, and polyethylene oxide resulted in a thicker layer of fibrosis around the implant comparing to thinner ones.^[^ ^124^ ^]^ In another study polypropylene fibers were implanted, with diameters ranging from 2.1 to 26.7 µm, in the subcutaneous dorsum of rats. At 5 weeks, increased capsule thickness was observed with a larger diameter of polymer fibers. The macrophage density with polymer fiber of 2.1 to 5.9 µm was comparable to that of the intact control group. For fibers with diameters in the ranges of 6.5 to 26.7 µm, increased macrophage densities were detected, all of which were greater than that of the control group. It was hypothesized that small fibers resulted in reduced cell‐material contact surface area to trigger cell signaling and led to reduced fibrous capsule thickness and macrophage density.^[^ ^125^ ^]^

Besides the shape, matrix architecture can also bring a significant influence on the macrophage. When bone marrow‐derived macrophages were seeded on electrospun polydioxanone scaffolds with various pore sizes and porosity, the cellular behavior depended on scaffold morphology.^[^ ^126^ ^]^ Larger fiber/pore size, which was electrospun from higher concentration solutions, led to increased expression of Arginase 1, and secretion of angiogenic cytokines VEGF, TGF‐*β*1, and *β*FGF. Meanwhile, the decreased expression of inducible nitric oxide was observed. These results collectively demonstrated that larger architecture promoted anti‐inflammatory macrophages (M2 subtype) development and suppressed inflammatory macrophages. The underlying mechanism was speculated that larger dimension facilitated macrophage infiltration, and enabled a more natural and spread‐out morphology which is favored by M2 macrophages.

Surface properties of implants, including surface texture, roughness, and hydrophilicity, also play a vital role in the immune response. Macrophages displayed a distinct response to the surface with different roughness.^[^ ^127^ ^]^ For comparison, RAW 264.7 macrophages were seeded on rough and smooth epoxy substrates. An M2‐like phenotype on rough surfaces was observed, with upregulated MCP‐1 and MIP‐1*α* but a lower secretion of the M1‐associated chemokine IP‐10 relative to smooth surfaces. This result is consistent with another work on titanium materials which found that the smooth titanium induced inflammatory macrophage (M1) activation, as indicated with increased levels of cytokines IL‐1*β*, IL‐6, and TNF‐*α*. In comparison, rough titanium resulted in an anti‐inflammatory M2‐like phenotype characterized by the secretion of IL‐4 and IL‐10.^[^ ^128^ ^]^ Besides, it was reported that rough titanium surfaces were preferred by monocytes compared to turned titanium surfaces.^[^ ^129^ ^]^ Furthermore, the patterned surface can be used to tune the macrophage phenotype through the elongation of adhering cells.^[^ ^130^ ^]^ Titanium surfaces containing micro‐ and nanopatterned grooves drove macrophages toward an anti‐inflammatory, pro‐healing phenotype, which secreted the highest levels of IL‐10 on the intermediate groove. Hydrophilicity also has a significant impact on the immune response. The hydrophilic titanium surface can modulate the immune response that suppressed secretion of TNF, IL‐1*α*, and chemokine (C‐C motif) ligand 1 (CCL‐1) from macrophages, and the macrophage gene expression profile is directing the inflammatory process toward a less pro‐inflammatory macrophage phenotype. The less pro‐inflammatory macrophage phenotype on the hydrophilic titanium surface can influence downstream healing events via the macrophage releasate.^[^ ^131^ ^]^ Furthermore, hydrophilic titanium surface could promote adaptive immune response toward inflammation resolution and enhanced stem cell recruitment. Titanium implants with increased surface roughness and wettability can polarize the adaptive immune response toward a Th2, pro‐wound healing phenotype, which results a faster resolution of inflammation and increased stem cell recruitment on rough hydrophilic surfaces with macrophages present.^[^ ^132^ ^]^ Anti‐biofouling surface through modifying the surface wettability could be beneficial to isolating the biomaterials from immune system. Several studies have shown that the hydrophilic surfaces could change the surface protein adsorption.^[^ ^133^, ^134^ ^]^ Besides, slippery liquid‐infused porous surfaces (SLIPS) have been created in recent years by introducing liquid polymer lubricants into microstructured substrates.^[^ ^135^ ^]^ The SLIPS provided a low surface energy and significantly reduced the protein adsorption, which could develop consistent favorable performance in vivo to avoid the chronic inflammation.^[^ ^136^, ^137^ ^]^

Besides the topological structure, material stiffness can also influence the cellular behavior of macrophage. Macrophages traveled faster on stiffer substrates and showed an enhanced proliferation rate,^[^ ^138^ ^]^ while soft substrate resulted in less active macrophages and reduced foreign body response.^[^ ^139^ ^]^ A similar result showed that macrophages grown on soft substrates produced less proinflammatory cytokines than those grown on stiff substrates.^[^ ^140^ ^]^ Soft surface can result in less focal adhesion of macrophages and impact macrophage activation through mechanomodulation mechanism,^[^ ^141^ ^]^ Soft substrate attenuates the inflammatory activity of macrophages, enhancing peroxisome proliferator‐activated receptor *γ* expression that suppressed M1 macrophage activity, and consequently promoting macrophages toward M2‐like phenotype. These findings indicate stiffness plays an important role in regulating the transition between monocyte phenotypes.

There are also a few studies using physical signals to modulate other immune cells, such as, T cells and dendritic cells. It was reported that substrates with modulus smaller than 100 kPa stimulated much more IL‐2 secretion and proliferation of human CD4+ and CD8+ T cells compared with stiffer substrates.^[^ ^142^ ^]^ On softer substrates, naive CD4+ T cells were more prone to expanded toward Th1‐like cells that produced cytokine IFN‐*γ*. Dendritic cells were found sensitive to surface charge density of liposomes.^[^ ^143^ ^]^ High charge density enhanced dendritic cell maturation and IFN‐*γ* secretion. In contrast, low‐charge liposomes failed to promote immune responses.

### 3.2 Chemical Signals

**Figure 4:**
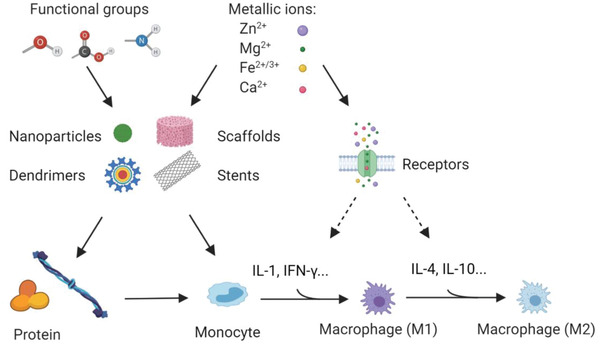
Chemical signals on the immunomodulation of biomaterials. Metallic ions could be released from biodegradable implants, including metallic scaffolds or stents, while functional groups could be released from biodegradable organic biomaterials and used for modification on nanoparticles, dendrimers, or scaffolds. Metallic ions and functional groups interact with protein or immune cell adhesion and play significant roles in the cytokines production or release in the immunomodulation process.

The chemical composition of implants has been considered another important factor to induce or impact foreign body response. Various inorganic signals (metallic ions, ceramics) and functional groups from metallic, ceramic, and organic biomaterials have been utilized to control interactions between implants and the immune system for immunomodulation (**Figure** **4**).

Metallic biomaterials have been historically developed as medical implants, for example, scaffolds and stents, to provide mechanical support for the injured tissue in reconstructive surgery.^[^ ^144^ ^]^ Biodegradable metals could degrade gradually in vivo to avoid a secondary removal surgery and have been regarded as promising candidates to the classic metallic biomaterials in orthopedic and cardiovascular applications.^[^ ^145^, ^146^, ^147^ ^]^ As the three representative biodegradable metals, zinc, magnesium (Mg), and iron have been studied in the past decades. The corresponding metallic ions released from them are discussed in terms of their biological roles in the immune system. It is noteworthy that the surface roughness and wetting property as discussed above could also affect the biodegradation rate and the metallic ions release from these implants.^[^ ^148^, ^149^ ^]^ In addition to the ion release from metallic implants, the ions have been used as a doping element in the immunomodulation of various organic, ceramic, and metallic biomaterials.^[^ ^150^, ^151^, ^152^ ^]^

Zinc, one of the trace elements to keep organ function normally, is well‐known as a second messenger in the immune system.^[^ ^153^, ^154^ ^]^ Zinc is found to be involved in many signal pathways and can activate both innate and adaptive immune cells through different peptides and receptors.^[^ ^145^, ^154^ ^]^ Depending on the time scale of their immune function occurs, the intracellular zinc signals can be classified as zinc flux (seconds to minutes), zinc wave (minutes), and homeostatic zinc signals (hours).^[^ ^155^ ^]^ Zinc regulation through metallothionein (MT) within innate immune cells, such as macrophages, is critical for cytokine production and antibacterial performance.^[^ ^156^ ^]^ Zinc concentration is significant in its interaction with the immune systems. It could induce pro‐inflammatory responses at low concentration while anti‐inflammatory responses at high concentration.^[^ ^157^, ^158^ ^]^ The higher level of zinc led to enhanced secretion of IFN‐*γ*, interleukin 12 receptor subunit (IL12R*β*2), and T‐bet to promote Th1 differentiation.^[^ ^159^ ^]^ Besides, the magnetron sputtering zinc incorporated surface could induce anti‐inflammatory responses for sulfonated polyetheretherketone as implant material in orthopedic applications.^[^ ^151^ ^]^ A Zn‐doped TiO_2_ nanotube on titanium (Ti) implants could also inhibit the pro‐inflammatory reactions and enhance the pro‐regenerative activities and thus promote the new bone formation.^[^ ^160^ ^]^

Mg is another critical element in many immune activities, such as, immunoglobulin synthesis, immune cell adherence, antibody‐dependent cytolysis, and macrophage response to lymphokines.^[^ ^161^ ^]^ It has been reported to reduce the production of TNF‐*α* and IL‐6 from monocytes, which indicates that it could decrease inflammatory reaction from innate immune cells.^[^ ^162^ ^]^ Mg‐doped calcium phosphate promoted TGF‐*β*1 secretion and suppressed the production of inflammatory cytokines including TNF‐*α* and IL‐6, while the Mg concentration in the surface had little influence.^[^ ^150^ ^]^ Ti implants doped with Mg (0.1–0.35%) by plasma implantation method could promote macrophages to polarize toward M2 phenotype with increased production of anti‐inflammatory cytokines IL‐4 and IL‐10 together with the up‐regulation of bone morphogenetic protein‐2 (BMP‐2) and VEGF when applied as implantable medical devices.^[^ ^152^ ^]^

Iron plays a significant role in modulating immune effector mechanisms in many immune cells, including lymphocytes, NK cells, T cells, monocytes, and macrophages.^[^ ^163^, ^164^ ^]^ Iron has also been reported to impact the immune system and disorder the iron metabolism leading to abnormal immune response.^[^ ^165^ ^]^ Although there are few studies on the iron ion related immunomodulation, the surface oxidation rate could be potentially modulated with the surface roughness and iron oxide has been used to modulate mice immune response of Th2 and the allergic response was suppressed or enhanced after treated with different particle doses and sizes.^[^ ^166^ ^]^

In addition to these three metallic ions, calcium (Ca) released from calcium‐based ceramic biomaterials has been used to modulate immune cellular function for tissue engineering, especially in musculoskeletal applications.^[^ ^167^, ^168^ ^]^ Calcium phosphate (CaP) ceramic biomaterials are widely used as bone substitutes or surface coatings on metallic implants through various chemical or physical methods,^[^ ^136^, ^169^, ^170^, ^171^ ^]^ but it is necessary to utilize the modification of metallic ions (zinc or strontium) or other ceramics to potentially reduce the acute and chronic inflammation associated with the CaP particles.^[^ ^172^, ^173^ ^]^ Calcium silicate (Ca_2_SiO_4_) has been shown as one of the ceramic biomaterials to sequentially activate macrophage polarization and thus modulate the vascularization of tissue engineering bone.^[^ ^174^ ^]^

Tons of organic biomaterials have been developed to mitigate the foreign‐body response and enhance engraftment and modify the surface chemistry of other biomaterials, especially the dendrimers and nano‐materials owing to their high surface area to volume ratio.^[^ ^175^ ^]^ Organic functional groups, including hydroxyl (—OH), carboxyl (—COOH), amine (—NH_2_), sulfhydryl (—SH), and phosphoryl (—PO_3_) groups, are critical in various biological functions of organic biomaterials.^[^ ^176^ ^]^

Hydroxyl is one of the most relevant forms of ROS and could induce immune response through many abundant regulated pathways, including migration inhibitory factor (MIF)‐Jun activation domain binding protein 1(MIF‐JAB1) and IL3 signaling, MIF‐mediated glucocorticoid regulation, oncostatin M signaling, and antigen presentation.^[^ ^177^ ^]^ Therefore, the hydroxyl group has been used universally to modify the surface chemistry of nanomaterials and organic biomaterials. The hydroxyl end group on the nanoparticles was identified as one of the key factors to activate DCs through a pathogen‐mimicking behavior.^[^ ^178^, ^179^, ^180^ ^]^ Hydroxyl functionalized carbon nanotubes showed higher cell viability while inducing the IL‐8 release and modulate antiviral and inflammatory immune responses.^[^ ^181^ ^]^ Hydroxyl poly(ethylene glycol) (PEG) has been reported with similar immunomodulation effects to decrease the immunogenic and antigenic effects of the methoxy PEG in the PEGylated protein therapeutics.^[^ ^182^ ^]^

Carboxyl has been used in the modification of nanomaterials and shown to suppress the immune response of macrophages.^[^ ^183^, ^184^ ^]^ It could significantly inhibit the M2 polarization on the mesoporous silica nanorods, as characterized by the decreased surface markers CD200R and CD163 and IL‐10 secretion. However, it helped the protein synthesis and TGF‐*β*1 secretion by M1 macrophages. This is beneficial to modulate the compromised immune response in cancer treatments.^[^ ^184^ ^]^ Another example is that the nano‐fibrillated cellulose (NFC) could induce an inflammatory response from macrophages, which is characterized by increased production of TNF‐*α* and IL1‐*β*. However, NFC modified with carboxyl groups resulted in much less inflammatory cytokines and hydroxypropyl trimethylammonium even eluted the immune response.^[^ ^185^ ^]^

Amine group on nanomaterials are favorable for complement activation and immune responses and could increase the Th2‐biased responses.^[^ ^186^, ^187^ ^]^ It has been reported that the surface after amine or acrylic acid treatment significantly modulated the osteoimmune response, which resulted in more inflammatory cytokine secretion of TNF‐*β*, IL‐18, and IL‐6 from macrophages.^[^ ^188^ ^]^ The underneath mechanism is that amine groups favor the absorption of fibrinogen, fibronectin, and albumin and enhanced the attachment of immune cells and subsequent immune response. However, the immune response could be related to the concentration of amine treatment,^[^ ^189^ ^]^ and it was also found to decrease phagocytosis by M1 and M2 macrophages on the mesoporous silica nanorods.^[^ ^184^ ^]^

In addition to the above functional groups, aldehyde could bind to the amino‐terminal end of peptide fragments, which induced the innate immune responses and also facilitate their recognition by the adaptive immune system.^[^ ^190^, ^191^ ^]^ Sulfhydryl on the biomaterial surface could provide the site‐specific and oriented conjugation of proteins and peptides, which induced the antitumor immunity against lymphomas.^[^ ^192^, ^193^ ^]^ Similarly, phosphoryl functional group on dendrimer could target the lymph node and be recognized by the phagocytes and B cells.^[^ ^194^ ^]^ Glycosaminoglycans like hyaluronic acid (HA) and heparin coated on solid slides could reduce nuclear factor kappa‐light‐chain‐enhancer of activated B cells (NF‐*κ*B) level to afford an anti‐inflammatory surface.^[^ ^195^ ^]^

### 3.3 Biological Signals

**Figure 5:**
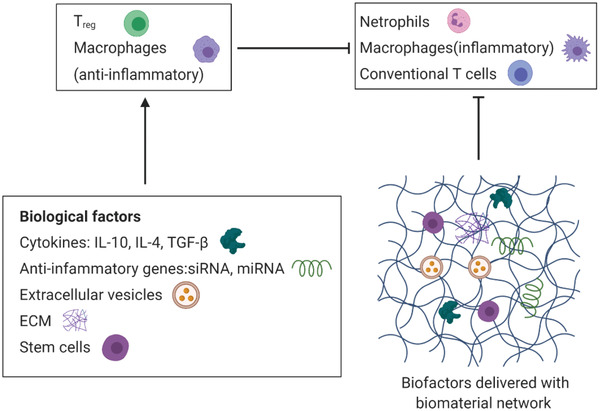
Immunomodulation with virous bioagents including cytokines, genes, extracellular materials (ECM), and stem cells. Biomaterial network is used as delivery vehicle and supporting scaffold for cell attachment, proliferation and differentiation.

Different from physical and chemical signals, using biological factors has been considered a straightforward way to regulate immune responses and usually plays as a more effective way for immunomodulation. Various biological factors such as cytokines, genes, and extracellular materials have been applied to regulate inflammation and promote tissue homeostasis (**Figure** **5**). Stem cells are also used for immunomodulation for biomedical application, but they are not a focus in this paper and can be referred to some excellent reviews elsewhere.^[^ ^196^, ^197^, ^198^ ^]^

As mentioned before, cytokines such as TNF‐*α*, IL‐6, and IFN‐*γ* promote inflammation, while IL‐4, IL‐10, and IL‐13 led to inflammation homeostasis. Therefore, in order to control inflammatory response, one strategy is to block inflammatory cytokines. Monoclonal antibody was used to treat transgenic mice which produce human TNF‐*α*.^[^ ^199^ ^]^ Significantly reduced arthritis was observed after anti‐TNF‐*α* treatment and loss of body weight was prevented due to suppression of circulating TNF‐*α* and local expression of various proinflammatory mediators. Decreased inflammation, necrosis, actin expression and fibrosis were observed in rat after treatment with antibody of TNF‐*α*.^[^ ^200^ ^]^ NF‐*κ*B pathway is considered an important role in the development of immune response and could impair healing of some tissues.^[^ ^201^ ^]^ Many efforts have been tried to downregulate NF‐*κ*B responses to help tissue regeneration. Through inhibition of endogenous inhibitor of *κ*B kinase (IKK)‐NF‐*κ*B in osteoblast, increased trabecular bone mass and bone mineral density were found in young mouse.^[^ ^202^ ^]^ Meanwhile, inhibition of IKK‐NF‐*κ*B led to enhanced expression of Fos‐related antigen‐1, which is involved in bone matrix formation. Different from suppression of pro‐inflammatory factors, direct application of anti‐inflammatory cytokines helps tissue homeostasis. Delivery of IL‐4 by osmotic pumps was found to promote mouse M0 and M1 macrophages to change toward M2 phenotype.^[^ ^203^ ^]^ IL‐10 was used to reduce inflammation and promote cardiac wound healing.^[^ ^204^ ^]^ Other biological factors such as genes and extracellular vesicles have also been explored for immune modulation. An anti‐inflammatory small interfering RNA was delivered by poly(lactic‐*co*‐glycolic acid) (PLGA) microparticles using a rat mode with temporomandibular joint inflammation, and reduced inflammatory cytokines were observed.^[^ ^205^ ^]^ IL‐10 lentivirus delivery to macrophages reduced NF‐*κ*B activation and TNF‐*α* production, promoting macrophages to polarize toward an M2 phenotype.^[^ ^206^ ^]^ Exosomes from *Leishmania donovani* were found to suppress immune reaction of human monocyte by promoting IL‐10 production and inhibiting that of TNF‐*α*.^[^ ^207^ ^]^

In order to protect biological agents and achieve better immunomodulation performance, implants/scaffolds have been used as localized drug delivery vehicle. Meanwhile, they can serve as ECM to preserve tissue architecture, support cell activities and promote tissue regeneration. Incorporated with immune regulators, there are several types of implants used in tissue engineering medicine, such as non‐resorbable metals/polymers, synthetic and natural resorbable polymers, as well as, their composites.

Titanium and its alloys are widely used as orthopedic implants due to their excellent corrosion resistance and biocompatibility. To improve metal and tissue integration and reduce inflammation, IL‐4/polydopamine was coated on the surface of titanium alloy, promoting macrophage to switch to M2 phenotype, and improved the in vivo metal implant‐soft tissue integration.^[^ ^208^ ^]^ In another study, titanium substrate was coated with IL‐4/graphene oxide and increased M2 macrophages were observed, leading to improved stability, bone‐implant contact and osteogenesis.^[^ ^209^ ^]^ In addition to single cytokine, sequential release of immunomodulatory mediators was applied to better control immune response.^[^ ^210^, ^211^ ^]^ Titanium implants containing IL‐4 and IFN‐*γ* that were encapsulated in PLGA and sodium hyaluronate, respectively, showed sequential release of loaded pro‐inflammatory and anti‐inflammatory cytokines.^[^ ^210^ ^]^ In another double release system, anti‐inflammatory cytokine IL‐4 was loaded in titania nanotube, and pro‐inflammatory cytokine IFN‐*γ* was located between two hydrogel layers that coated on titania substrate.^[^ ^211^ ^]^ IFN‐*γ* showed a rapid release, whereas IL‐4 exhibited a sustained release profile. As a result, macrophages were first polarized toward M1 that was stimulated by IFN‐*γ* and subsequently switched to M2 phenotype triggered by IL‐4 release. Metal materials have much higher mechanical strength than that of bone, causing stress shielding issue. Meanwhile, ions released from metallic implants could be toxic. To overcome these disadvantages, non‐degradable polymers have been chosen as an alternative of permanent implants for orthopedic application. Polyethylene (PE) is frequently used for hip and knee arthroplasty.^[^ ^212^, ^213^ ^]^ Local delivery of IL‐4 on PE particles implanted in mice skull resulted in reduced M1/M2 ratio of macrophage and bone loss decreased.^[^ ^214^ ^]^ Vitamin E was diffused into highly cross‐linked ultra‐high molecular weight polyethylene (UHMWPE) particles to combat inflammatory reaction and less TNF‐*α* secretion from macrophage was found when exposed to these particles.^[^ ^215^ ^]^ Polypropylene (PP) is another type of polymer used for prosthetic devices.^[^ ^216^ ^]^ To improve loading efficacy, IL‐4 was loaded into a nanometer thickness of chitosan/dermatan sulfate matrix coated on plasma‐treated PP mesh,^[^ ^217^ ^]^ and increased M2 macrophages along with decreased M1 macrophages at the tissue‐implant interface were observed.

Comparing to non‐resorbable metallic and polymer materials, resorbable polymers possess advantages such as better affinity with cytokines and tunable degradation rate to match tissue regeneration. Poly(lactic acid) (PLA) is one of the most used synthetic degradable polymers in tissue regeneration medicine.^[^ ^218^, ^219^ ^]^ Using 3D printing technique, porous PLA scaffold was fabricated.^[^ ^220^ ^]^ Through incorporation with chitosan and coating with polydopamine layer, bioactive quercetin was loaded onto this scaffold to promote osteogenic activity and reduce inflammation of MC3T3‐E1 cells. Comparing to hydrophobic PLA, PLGA is more hydrophilic and has advantages such as better cell affinity and adjustable degradation behavior through change of lactic/glycolic ratio.^[^ ^221^ ^]^ Using salt‐leaching process, porous PLGA scaffolds were prepared after sodium chloride particles being washed away from PLGA/NaCl mixture.^[^ ^222^ ^]^ These PLGA scaffolds incorporated with stromal cell‐derived factor (SDF)‐1*α* through adsorption and mini‐pump delivery resulted in increased stem cell population, reduced mast cell populations and degranulated mast cells around implants, subsequently leading to reduced inflammatory reaction and fibrotic response to scaffold implants. Combining gas‐foaming method with carbon dioxide and salt‐leaching processing with sodium chloride particles, PLGA porous scaffolds showed a high loading capacity of resveratrol and biphasic drug release behavior.^[^ ^223^ ^]^ After implantation of PLGA scaffolds into mouse adipose tissue, higher arginase‐1, and lower TNF‐*α* and IL‐6 expressions were detected. This anti‐inflammatory environment was further enhanced by resveratrol release, with decreased monocyte and lymphocyte populations at the implant site and increased secretion of IL‐10 and IL‐13 cytokines. Electrospinning technique is commonly used to fabricate nano/micro porous mesh.^[^ ^220^, ^224^ ^]^ PLGA incorporated with ibuprofen could be electrospun into a uniform fibrous mesh, and Ibuprofen showed a rapid release in the first few hours followed by slower release over several days.^[^ ^220^ ^]^ The released Ibuprofen didn't affect fibroblast attachment and proliferation on scaffolds and significantly reduced its response to major pro‐inflammatory stimulators. PEG is a versatile and highly hydrophilic polymer for medical application.^[^ ^225^, ^226^ ^]^ Hydrogel based on star‐shaped PEG was prepared through chemical crosslink with heparin, showing a sustained release of IL‐4 over two weeks and promoting macrophages toward M2 phenotype.^[^ ^227^ ^]^ A dual anti‐inflammatory drug delivery system based on PEG was found to promote immune cells toward pro‐regenerative phenotypes.^[^ ^228^ ^]^ This hydrogel was crosslinked by cleavable crosslinkers, and loaded with IL‐10 and aspirin‐triggered resolvin‐D1. The release of anti‐inflammatory drugs localized immune suppressive subsets including CD206+ macrophages and IL‐10 expressing dendritic cells to the hydrogel.

Natural polymers have superior biosafety that are ideal candidates to serve as ECM to delivery cytokines and support cell activities. HA is a polymer of glycosaminoglycans and can maintain tissue moist.^[^ ^229^ ^]^ In a HA gel system that formed through guest‐host interactions by adamantane (Ad) and *β*‐cyclodextrin, IL‐10 was loaded into HA hydrogel scaffold and injected subcutaneously in mice.^[^ ^230^ ^]^ IL‐10 showed a sustained and local release from HA hydrogel, leading to reduced renal and systemic inflammation. To increase binding ability to cytokine IL‐10, hydrogel composed of HA and heparin was crosslinked by PEG diacrylate to deliver IL‐10.^[^ ^231^ ^]^ This system was found significantly more effective than free IL‐10 to prevent and reduce collagen deposition in the lung parenchyma. A photoresponsive HA‐based hydrogel using ultraviolet (UV) light to conjugate Arg–Gly–Asp (RGD) peptide onto HA hydrogel scaffold could activate *α*v*β*3 integrin macrophage expressions, resulting in enhanced anti‐inflammatory M2 macrophage polarization.^[^ ^232^ ^]^ Collagen is the main structural protein in various tissues.^[^ ^233^ ^]^ A collagen scaffold functionalized with chondroitin sulfate (CSCL) was prepared through crosslink with 1,4‐butanediol diglycidyl ether in solution, followed by lyophilization.^[^ ^234^ ^]^ Upregulation of anti‐inflammatory markers and decrease in the expression of pro‐inflammatory markers from macrophages were observed with CSCL scaffold treatment. In a rat model, CSCL was implanted subcutaneously, resulting in a significant downregulation of pro‐inflammatory genes expression. To improve mechanical strength, collagen scaffold was incorporated with PLGA and silicon particles.^[^ ^235^ ^]^ Through a double emulsion method, IL‐4 was loaded to this scaffold. In vitro experiment results demonstrated that anti‐inflammatory associated genes from macrophages increased. Gelatin is commonly derived from collagen.^[^ ^236^ ^]^ Gelatin sponge loaded with BMP‐2 dramatically reduced the expression of M1 macrophage markers, including IL‐1β, IL‐6, and inducible nitric oxide synthase (iNOS).^[^ ^237^ ^]^ Silk is a natural protein fiber mainly composed of fibroin.^[^ ^238^ ^]^ 3D silk scaffolds prepared by freeze‐drying afforded them high porosity and well‐connected macropores.^[^ ^239^ ^]^ Incorporation of nicotinic acid into silk scaffolds showed a sustained drug release and suppressed gene expression of pro‐inflammatory markers TNF‐*α*, CXCL10, and CD197 from macrophages. A silk hydrogel system formed by simply blending mulberry and non‐mulberry silk.^[^ ^240^ ^]^ IL‐4 and Dexamethasone released from silk hydrogel polarized macrophages toward M2 phonotype. Chitosan is a linear polysaccharide mainly from crustaceans.^[^ ^241^ ^]^ Porous chitosan scaffold was prepared by lyophilization of degassed chitosan solutions in acid condition and Resolvin D1 (RvD1) was loaded onto the porous scaffold.^[^ ^242^ ^]^ Using a mouse air‐pouch model, RvD1 loaded scaffold resulted in decreased population of M1 macrophages and increased number of M2 macrophages. IFN‐*γ* and IL‐4 cytokines were loaded on decellularized bone scaffold through physical adsorption and biotin‐streptavidin binding, respectively, leading to sequential delivery of these two cytokines.^[^ ^243^ ^]^ IFN‐*γ* showed a short release, followed by a sustained IL‐4 release due to the stronger interaction with bone scaffold, which resulted in sequential M1 and M2 polarization of macrophages.

Besides aforementioned materials, some scaffolds made of bioactive ceramics/minerals were reported. IFN‐*γ* loaded on 3D printed calcium silicate‐*β*‐tricalcium phosphate scaffold was used to sequentially activates macrophages for vascularization.^[^ ^174^ ^]^ As discussed, permanent metallic and polymer implants have the best mechanical strength as prostheses, but their interaction with cytokines is weak and usually need the help of ceramics/minerals and hydrophilic polymers to enhance the loading the efficacy. Resorbable synthetic scaffolds have moderate mechanical strength and interaction with biological factors, as well as various available fabrication methods. Natural polymer scaffolds usually have the best biosafety, high interaction with cytokines, and low strength mechanical properties.

## 4 Tissue Regeneration/Repair Regulated with Immunomodulation

**Figure 6:**
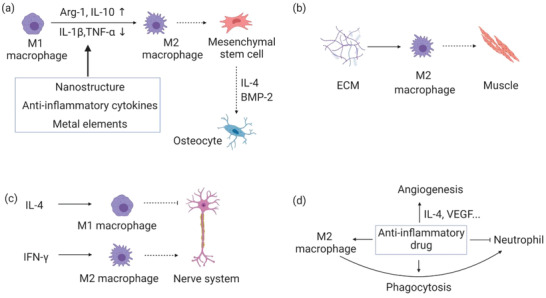
Different tissue regeneration through immunomodulation by various regulators. a) Bone regeneration with the help of physical, chemical, and cytokines. b) Muscle regeneration with the help of ECM. c) Nerve system regeneration with the help of cytokines. d) Vessel regeneration with the help of drugs. Arg‐1: Arginase‐1; BMP‐2: Bone morphogenetic protein 2.

The immune system has deeply and intensively participated in tissue regeneration. As a result, it is crucially important to manipulate immune processes to achieve a more efficient tissue healing outcome to establish a new best clinical practice. We seek to obtain this goal, through suppression of detrimental immune response and promotion of tissue restoration. Many efforts have been tried to promote tissue regeneration such as bone tissue (**Figure** **6a**), muscle (Figure 6b), nerve system (Figure 6c), and blood vessel (Figure 6d) with the help of immune regulatory factors delivered with bio‐scaffolds.

### 4.1 Skeletal Tissue Regeneration/Repair

Implants/scaffolds such as, non‐degradable metals, bio‐glasses/ceramics, resorbable synthetic, and natural polymers are used to deliver immunomodulatory signals for bone and cartilage regeneration. These signals include metal/inorganic ions, biomolecules such as polymers, macromolecules, and ECM, as well as, material physical properties. To fabricate implants/materials, various engineering methods are applied. Surface grafting is used for metal modification. Sol–gel processing followed by sintering is to prepare bioglass/ceramic scaffolds. Crosslinking is used for hydrogel preparation. Mineralization is applied for hybrid scaffold fabrication. 3D printing is suitable for all kinds of materials using relevant printing method.

Zymosan, which is a fungal component, was coated onto titanium substrate to promote bone formation and integration with metal implants.^[^ ^244^ ^]^ Titanium substrate was cleaned by polish and solvent washing. After activated with “piranha solution,” titanium surface was grafted with zymosan modified with reactive chemical groups for grafting zymosan. As a consequence of polarization of macrophages toward to pro‐regenerative stimulated by zymosan, a larger number of osteogenic/anioenic cytokines were observed in vitro study. In a rat femur condyle defect model, zymosan‐coated titanium substrate resulted in a remarkable increase in bone mineral density and bone volume, as well as improved bone‐implant integration. Magnesium/zinc coating on titanium implants could both inhibit bacterial infection and promote bone regeneration.^[^ ^245^ ^]^ After cleaning, titanium substrate was coated with magnesium/zinc metal organic framework after immersion in different metal salt solutions followed by high‐temperature treatment. Comparing to unmodified titanium substrates, increased secretion of anti‐inflammatory mRNAs and decreased inflammatory genes from macrophages were observed with magnesium/zinc coated substrates. Consistent with in vitro data, magnesium/zinc coated titanium substrates reduced inflammation locally after implantation into rat femur medullary cavity, and significantly improved new bone formation. Incorporated with niobium, porous titanium scaffolds fabricated with 3D printing technique using laser melting method showed high capacity to improve bone regeneration.^[^ ^246^ ^]^ The addition of niobium increased hydrophilicity of titanium scaffolds, activated anti‐inflammatory macrophages, and promoted osteogenesis. After implantation into rabbit femoral condyle defects, these niobium/titanium scaffolds showed increased new bone formation and enhanced bone‐implant contact.

Bioactive glasses, ceramics and minerals are resorbable materials possessing high mechanical strength suitable for skeletal tissue regeneration. Copper‐incorporated bioactive glass‐ceramics were prepared through sol–gel method,^[^ ^247^ ^]^ which then 3D printed into mesh‐like structures followed by high temperature sintering. The copper‐contained scaffolds drove macrophages toward an anti‐inflammatory phenotype and promoted proliferation of chondrocytes. Using rabbit osteochondral defect model, improved regeneration of cartilage and the recovery of the osteochondral interface were observed. Strontium‐substituted sub‐micrometer bioactive glass (Sr‐SBG) was also reported to promote bone regeneration.^[^ ^248^ ^]^ In vitro study demonstrated Sr‐SBG promoted osteogenesis of mouse mesenchymal stem cells and suppressed osteoclastogenesis of RAW 264.7 cells through proper modulation of inflammatory response. Sr−SBG was synthesized by an alkali‐catalyzed sol−gel method followed by sintering. In rat femoral condyle defect model, Sr‐SBG animal group showed a less immune response and improved bone regeneration comparing to SBG control group. Loaded with gold nanoparticles, mesoporous silica was observed to promote bone regeneration through immune regulation.^[^ ^249^ ^]^ The gold‐silica composites could stimulate an anti‐inflammatory response from macrophages, subsequently promoted osteogenesis. Furthermore, accelerated new bone formation in a critical‐sized cranial defect was observed with chitosan loaded with gold‐silica nanoparticles.

Synthetic polymers have less mechanical strength and excellent processing performance. A 3D printed PLGA scaffold, which was decorated with ECM derived from human umbilical cord mesenchymal stem cells (HUCMSCs), showed immunomodulation capacity for bone regeneration.^[^ ^250^ ^]^ HUCMSCs were seeded and cultured on 3D printed PLGA porous scaffolds, followed by decellularization. These PLGA‐ECM scaffolds increased population of M2 macrophages and improved bone regeneration in a mice femur defect model. A hybrid system composed of poly(*ε*‐caprolactone) (PCL)/nano‐hydroxyapatite porous scaffold incorporated with chitin‐derived hydrogels improved osteogenesis and angiogenesis in rat calvarial defect model.^[^ ^251^ ^]^ MSCs were encapsulated in chitin hydrogel and helped to activate M2 macrophages, leading to improved bone repair.

Scaffolds made of silicified collagen (SCSs), which was produced by incorporating collagen matrices with amorphous silica, was reported to promote bone tissue repair through monocyte immunomodulation.^[^ ^252^ ^]^ In a mouse calvarial defect model, sustained release of silicic chemicals stimulated monocytes differentiation, and increased secretion of SDF‐1*α*, TGF‐*β*1, and VEGF*α*, promoting neovascularization of bone tissue. A collagen/resveratrol scaffold showed anti‐inflammatory capacity and was used for rabbit osteochondral defect repair.^[^ ^253^ ^]^ Resveratrol was grafted to polyacrylic acid, followed by incorporation into atelocollagen hydrogel. In vivo study demonstrated this scaffold exhibited the capacity to reduce inflammatory reaction and promote bone and cartilage regeneration. A biomimetic hierarchical intrafibrillarly mineralized collagen (HIMC) with a bone‐like staggered nanointerface has been investigated for its immunomodulatory properties for bone regeneration.^[^ ^254^ ^]^ HIMC was prepared using mineralization process followed by lyophilization to afford 3D sponge‐like scaffolds. This scaffold facilitated macrophage to polarize toward M2 phenotype and stimulate IL‐4 secretion to enhance MSC osteogenesis, promoting endogenous bone regeneration in rat mandible defect model.

Gelatin nanofibers, which was modified with heparin and incorporated with IL‐4, resulted in accelerated bone regeneration in diabetes mellitus.^[^ ^255^ ^]^ This kind of scaffold was prepared through emulsification technique and phase separation process. Heparin can bind with IL‐4 and protect it from denaturation and degradation, enabling sustained released of IL‐4 and subsequently driving proinflammatory M1 macrophage switching to anti‐inflammatory M2 phenotype. In a rat mandibular periodontal fenestration defect model, improved bone regeneration was observed in animal group implanted with IL‐4‐loaded scaffold. 3D bioprinted hydrogel scaffolds composed of gelatin, PEG and silica nanoparticles, was loaded with bone morphogenic protein‐4 (BMP‐4), promoting polarization of macrophages toward M2 phenotype.^[^ ^256^ ^]^ In calvarial critical‐size defect models of diabetic rats, accelerated bone regeneration was found with hydrogel scaffolds containing BMP‐4, Bone MSCs, and RAW264.7 cells. Gelatin hydrogels that could release both SDF‐1 and BMP‐2 were found to enhance the recruitment of osteogenic cells and angiogenesis in a rat ulna critical‐sized defect.^[^ ^256^ ^]^ A biomimetic gelatin/fish bone hybrid hydrogel system was prepared by photopolymerization of gelatin methacrylate and nano fish bone powder.^[^ ^257^ ^]^ The incorporation of nano fish bone enhanced the mechanical strength of gelatin hydrogel and modulate the immune microenvironment to promote bone regeneration in rat craniotomies defects. 3D bioprinted hydrogel, which was composed of alginate incorporated with gelatin and hydroxyapatite nanoparticles, was loaded with Atsttrin, reducing the population of TNF‐*α* positive cells and enhancing bone regeneration in a mice calvarial bone defects.^[^ ^258^ ^]^

Bi‐layer porous scaffold, wherein a gelatin methacrylate scaffold printed with digital light processing as the upper layer and a PCL‐HAp scaffold printed with fused deposition modeling as the lower layer, was loaded with IL‐4 into gelatin layer.^[^ ^259^ ^]^ This hybrid scaffold reduced inflammation on murine chondrocytes and promoted regeneration of both cartilage and subchondral bone in a rabbit osteochondral defect model. A biomimetic gelatin hydrogel system, which was prepared through “chemical‐curing, shaping, and light‐curing” process, was used for cartilage repair.^[^ ^260^ ^]^ This hydrogel modulated pro‐inflammatory/anti‐inflammatory phenotypes of neutrophils and macrophages, and promoted chondrogenesis of cartilage stem/progenitor cells. In rabbit costal cartilage defect model, enhanced cartilage regeneration was observed. Thermosensitive hydrogels prepared by crosslinking of methacrylate‐modified chitin, was loaded with TGF‐*β*1,^[^ ^261^ ^]^ promoting M1 macrophage to polarize toward M2 phenotype and achieving superior cartilage healing in a rat cartilage defect. Scaffold composed of decellularized cartilage ECM and PEG diacrylate integrated with honokiol was prepared through stereolithography‐based 3D printing technique.^[^ ^262^ ^]^ In vitro data showed suppressed proinflammatory cytokines secreted from macrophages and improved new bony tissue formation was observed in a rat osteochondral defect.

### 4.2 Soft Tissue Regeneration/Repair

The underlying mechanism of soft tissue regeneration is to reduce anti‐inflammatory reaction, mainly switching macrophages to M2 phenotype, to promote tissue regeneration, which is similar as that for bone and cartilage repair. Different from regeneration of skeletal tissues, scaffolds used for soft tissue regeneration mostly have with low mechanical strength such as hydrogels and meshes, to match tissue mechanical properties. Lyophilization is a simple method to make powders, but scaffolds made of powders are usually brittle and easy to break. Physical and chemical crosslinking are two main methods to prepare hydrogels. One common physical crosslinking method is cooling solution from high temperature, which may denature biomolecules. Chemical crosslinking can be conducted at physiological conditions, but irradiation by light has penetration limitation.^[^ ^263^ ^]^ 3D printing is a rapid and customized technique to fabricate porous hydrogel scaffolds, but an extra step is commonly needed for solidification.^[^ ^264^ ^]^

Intestinal submucosa ECM bioscaffold was used to regulate immune response and repair volumetric muscle defect created in the region of mouse quadriceps muscle.^[^ ^265^ ^]^ The ECM scaffolds, which were prepared by lyophilization and milling, resulted in increased cellular infiltration and neomatrix deposition. Meanwhile, a large volume of M2‐type macrophages were observed at the defect site to promote muscle myotube formation. Decellularized cardiac tissue and MSCs could promote M2 macrophage development and suppress M1 formation, helping muscle regeneration in a rat tibialis anterior muscle defect model.^[^ ^266^ ^]^ Hydrogel based on type I collagen combining with MSCs was found to facilitate M2 macrophage transition, decrease collagen deposition and accelerate muscle repair with upregulated angiogenesis and myogenesis in rat volumetric muscle loss.^[^ ^267^ ^]^

An agarose gel loaded with IFN‐*γ* or IL‐4 could modulate macrophage phenotype to improve nerve repair.^[^ ^268^ ^]^ Both of M1 and M2 macrophages, which were induced by cytokines IFN‐*γ* and IL‐4, enhanced migration of Schwann cells. However, M1 macrophages slightly decreased proliferation of Schwann cells, whereas M2 phenotype did not. Implantation of this cytokine‐loaded hydrogel in a rat peripheral nerve defect demonstrated that the ratio of M2/M1 macrophages had a direct correlation with number of axons at the distal end of the nerve scaffold. Photo‐crosslinked gelatin hydrogel transplantation combined with colony stimulating factor 1 receptor inhibitor treatment was used to repair spinal cord injury, reducing reactive macrophages and expression of pro‐inflammatory genes, and leading to increased number of neurons.^[^ ^269^ ^]^ To increase hydrogel mechanical strength, PCL electrospun fiber was incorporated into PEG based hydrogel.^[^ ^270^ ^]^ In an adult rat model of spinal cord contusion treated with this strengthened hydrogel, higher M2/M1 macrophage ratio, axon density, immature neuron, and larger spinal cord segment were observed.

Scaffold based on decellularized bone tissue that could sequentially release IFN‐*γ* and IL‐4 was designed. This scaffold promoted polarization of macrophages toward M1 and M2 phenotypes, respectively.^[^ ^243^ ^]^ IFN‐*γ* led to increased M1 macrophages in the early stage, while IL‐4 increased M2 macrophage polarization. Subcutaneous implantation of scaffolds loaded with IFN‐*γ* and IL‐4 in a mouse animal model showed increased vascularization. Using layer‐by‐layer assembly method, heparin and selenium‐containing catalyst‐organoselenium modified polyethyleneimine were introduced to electrospun PCL mesh to form a bioactive vascular graft.^[^ ^271^ ^]^ After implantation to replace the rat abdominal aorta, the modified graft was found to promote M2 macrophage formation and enhance endothelialization. 3D microchannel networks based on a gelatin hydrogel rescued damaged tissues by ingrowth of neighboring host vessels in mouse and porcine models of hindlimb ischemia, which was guided by the regenerative macrophage polarization.^[^ ^272^ ^]^

Chitosan hydrogel incorporated with prostaglandin E_2_ was applied to repair cutaneous wound.^[^ ^273^ ^]^ The hydrogel showed prolonged release of prostaglandin E_2_, promoting the M2 macrophage transformation, and balancing inflammation, regeneration and remodeling during mouse cutaneous wound healing. Gelatin based adhesive hydrogel containing microRNAs was load with HA nanoparticles for mouse wound healing, promoting M2 macrophage formation that resulted in uniform vascularized skin at the wound site.^[^ ^274^ ^]^ Plasmid DNA encoding VEGF and resveratrol were loaded into hydrogels composed of HA, dextran, and *β*‐cyclodextrin, accelerating healing of rat splinted excisional burn wound through inhibition of inflammation response.^[^ ^275^ ^]^

## 5 Summary and Future Direction

The immune system plays a vital role in responding to tissue damage and subsequent tissue regeneration. Therefore, how to appropriately manipulate and govern the immune response to best harness the innate healing process is crucial for tissue restoration. By way of the strategies and methodology discussed in this work, a multitude of efforts have been made to explore a means to regulate behavior of immune cells through. Factors to do so include physical, chemical, and biological stimuli, all of which primarily focused on limiting inflammation reaction and promoting toward a regeneration phase. Neutrophils are usually the first responders after tissue injury and help to clear cell debris. Recent studies also indicated that neutrophils are involved in macrophage polarization and contribute to tissue regeneration. Macrophages have shown an important role during tissue repair and participate in multiple phases from inflammation to resolution stages, which involve pro‐inflammatory M1 macrophages and anti‐inflammatory M2 phenotype. M1 macrophages are considered detrimental to tissue repair. On the contrary, M2 macrophages have pro‐regenerative capacities which can be further divided into M2a, M2b, and M2c subtypes. However, persistent activation of M2a could lead to pathological fibrosis formation. Similar to macrophages, T cells also have several subtypes. Th2, Treg, and *γδ*T cells help to resolve inflammation and promote tissue regeneration.

Though much knowledge has been gained on immunomodulation and based on the knowledge, we can rationally design strategies to manipulate immune response to achieve better tissue regeneration, however, there is still more unknow need to explore. For example, neutrophils, which are traditionally considered to be detrimental to tissue repair, have shown a protective role to help resolve inflammation. However, the underling mechanism is not well studied. Macrophages have several subtypes involved in inflammation and subsequent regenerative process of damaged tissue. It remains unclear how these transitions between pro‐inflammatory and anti‐inflammatory phenotypes happen or whether they are simply subtle variants originating from monocytes. Similarly, conventional T cells are reported to impair tissue healing. On the contrary, regulatory T cells can modulate innate immune cells and conventional T cells to help tissue healing toward a regenerative pathway. Nevertheless, we still have sparse knowledge on how regulatory T cells control behaviors of other immune cells during tissue healing process. In general, immunomodulation for tissue regeneration involves very complicated physiological process. We should further explore the underlying mechanism and thus can better harness immune system to improve tissue repair.

As aforementioned, there are many different methods used to regulate immune response after tissue injury. However, with respect to clinical application, it is still a long way to go considering on biosafety and effectiveness. Biocompatible materials, especially those that have been approved by FDA, can be used to reduce the possible regulatory risk and help to facilitate clinical translation. On the other hand, to achieve precise modulation on the specific immune cells, targeted delivery strategies should be considered. Incorporation of guiding components that target specific cells or pathways into drug delivery vehicle (scaffolds) can increase therapeutic efficacy and simultaneously reduce side effect. Since immune response is a sequential multiple‐step and dynamic process, therefore, multi‐components that sequentially and dynamically activate distinct type of immune cells can be applied to achieve better tissue regeneration.

## Conflict of Interest

The authors declare no conflict of interest.
